# Psychiatrists Can Also Experience Epistemic Injustice: Reconnecting with Ethos in Mental Health

**DOI:** 10.5195/pom.2024.209

**Published:** 2024-10-01

**Authors:** Timothy Daly, Paola Buedo

**Affiliations:** 1 Science, Norms, Democracy, UMR 8011, Sorbonne University, Paris, France; Bioethics Program, FLACSO, Argentina, Buenos Aires, Argentina.; 2 Institute of History and Ethics in Medicine, Department of Preclinical Medicine, TUM School of Medicine and Health, Technical University of Munich, Munich, Germany; Bioethics Program, FLACSO Argentina, Buenos Aires, Argentina.

In their letter in *Philosophy of Medicine*—and other articles like it—Ian James Kidd, Lucienne Spencer, and Eleanor Harris provide a rich tool kit through which to understand epistemic injustice in psychiatry (Kidd, Spencer, and Harris 2023). They are right in saying that “we should not overlook the psychiatrist’s unique epistemic power, and the ways epistemic injustices in this domain can have serious consequences” (2023: 3). We wish to extend those consequences to mental health practitioners themselves. This reflection leads us to argue that the problem of epistemic injustice goes beyond the psychiatrist–patient relationship to encompass the relationships between practitioners, particularly when a colleague experiences mental health issues. When we talk about epistemic injustice toward people suffering from poor mental health, we should not limit ourselves to consideration of patients only.

Indeed, in a recent article, psychiatrist Dr. Anne Révah-Levy relates her experience of “stigma from mental health professionals” toward her own experience of mental illness (Sibeoni and Révah-Levy 2023: 834). Most of her colleagues could not “relate to and rationalize” her suffering, other than a few exceptions, including the coauthor of her article, who has a family member with bipolar disorder (2023: 834). Evidence shows that stigma toward mental illness is prevalent among medical students and professionals, including psychiatrists, and also that the presence of a relative with mental illness leads to reduced stigma (Henderson et al. 2014). Such stigma is paradoxical because of practitioners’ “knowledge and literacy about mental disorders” and their “value-based practice with their patients” (Sibeoni and Révah-Levy 2023: 834). Their recognition that reliance on a biological or systemic understanding of mental illness “does not fully explain the genuine and warm attitude of [the] few individuals” who treated Dr. Révah-Levy humanely (2023: 834) reminds us that historically the competence of a medical practitioner (*technikós*) was not reducible to literacy about diseases, but required the ability and willingness to know the patient and to apply medical technique to the uniqueness of that particular patient.

While we are comforted by Dr. Révah-Levy finding her voice in the book she wrote (Révah 2021), her experience is a reminder of the need to listen to healthcare service users and those who work within it, which is “not some politically correct or sentimental add-on to medical conversations. It is the essence of medical practice … the patient’s narrative … [is] an act of recovery and re-creation by the patient, with the doctor acting as facilitator” (Launer 2022: 236). Mental health professionals have a responsibility to create mentally healthy environments for patients and colleagues as part of their professional ethos, by exercising professional ethics and committing to overcoming their own biases. Importantly, strong paternalism was used to justify Dr. Révah-Levy’s exclusion from her work environment as in the phrase, “it is for your own sake and for the team” (Sibeoni and Révah-Levy 2023: 834), with tones of gender bias, since women with mental illness are perceived as less capable of making decisions in healthcare settings (Harbin 2023).

Initial and ongoing medical education should seek to provide resources to enable practitioners to achieve healthy environments. Miranda Fricker (2007) argues that hearers of testimony must overcome any propensity they might have to assign knowers with less credibility. This is of course particularly applicable to the psychiatrist–patient relationship (Crichton, Carel, and Kidd 2017). Within the study of mental health, the cultivation of person-centered values should be at least on a level footing with literacy about disorders. However, this mission might only be achieved with deliberate reflection from an ethical standpoint, requiring an overhaul of the concepts we use to think about mental health in ways that go beyond psychiatry (Buedo and Daly 2023).
